# Confinement and Polarity Effects on the Peptide Packing Density on Mesoporous Silica Nanoparticles

**DOI:** 10.1021/acs.langmuir.3c03513

**Published:** 2024-02-12

**Authors:** Bastian Beitzinger, Roman Schmid, Christoph Jung, Kanishka Tiwary, Patrick Hermann, Timo Jacob, Mika Lindén

**Affiliations:** † Institute of Inorganic Chemistry II, Ulm University, Albert-Einstein-Allee 11, Ulm 89081, Germany; ‡ Institute of Electrochemistry, Ulm University, Albert-Einstein-Allee 47, Ulm 89081, Germany; § Department of Internal Medicine I, Ulm University, Albert-Einstein-Allee 23, Ulm 89070, Germany

## Abstract

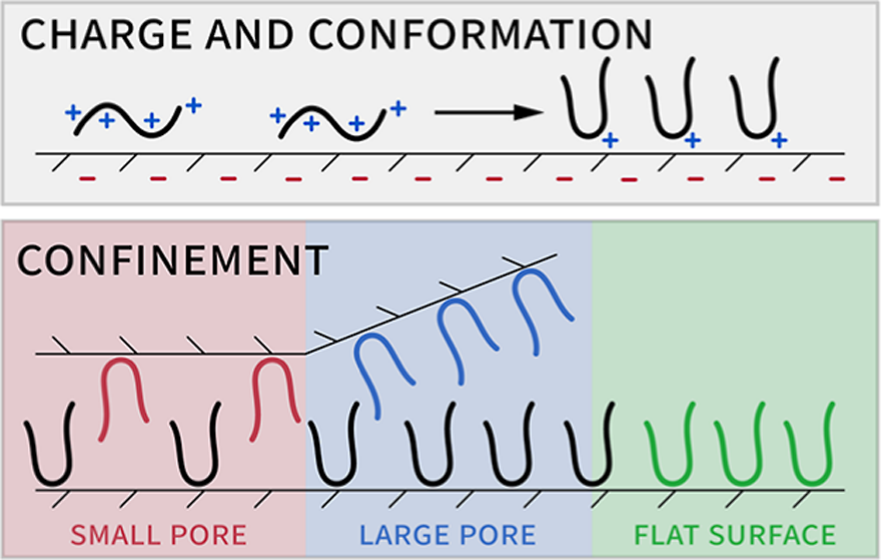

The adsorption of cationic peptide JM21 onto different mesoporous silica nanoparticles (MSNs) from an aqueous solution was studied as a function of pH. In agreement with the literature, the highest loading degrees could be achieved at pH close to the isoelectric point of the peptide where the peptide–peptide repulsion is minimum. However, mesopore size, mesopore geometry, and surface polarity all had an influence on the peptide adsorption in terms of both affinity and maximum loading at a given pH. This adsorption behavior could largely be explained by a combination of pH-dependent electrostatic interactions and confinement effects. It is demonstrated that hydrophobic interactions enhance the degree of peptide adsorption under pH conditions where the electrostatic attraction was absent in the case of mesoporous organosilica nanoparticles (MONs). The lower surface concentration of silanol groups for MON led to a lower level of peptide adsorption under optimum pH conditions compared to all-silica particles. Finally, the study confirmed the protective role of MSNs in preserving the biological activity of JM#21 against enzymatic degradation, even for large-pore MSNs, emphasizing their potential as nanocarriers for therapeutic peptides. By integrating experimental findings with theoretical modeling, this research elucidates the complex interplay of factors that influence peptide–silica interactions, providing vital insights for optimizing peptide loading and stabilization in biomedical applications.

## Introduction

The potential of mesoporous silica nanoparticles, MSNs, for applications within the biomedical domain has been extensively demonstrated, especially in relation to diagnostics, biosensing, and theranostics during the last two decades.^1−4^ Their high surface area and customizable pore system facilitate adsorption of various therapeutics ranging from small-molecular drugs to macromolecular entities like peptides, proteins, and nucleic acids.^5−9^ Particularly, peptide and protein encapsulation within MSNs has been demonstrated to give protection against enzymatic degradation, hence prolonging their functional viability.^10−13^

In the realm of drug delivery, the diverse physical and chemical properties of peptides and proteins necessitate a versatile carrier system for optimal loading and release profiles. Consequently, numerous studies have delved into understanding the various factors influencing peptide and protein adsorption onto silica-based materials.^8,14−17^ For ideally flat silica surfaces, this exploration essentially pivots on the interactions between the adsorbent and adsorptive, which can encompass van der Waals forces, hydrogen bonds, and notably, electrostatic forces—the strongest short-range forces in this context.^18^ Surface silanol groups of amorphous silica have p*K*_a_ values in the range of about 4.5–8.5, and consequently, the silica surface is negatively charged over a broad pH range. Therefore, electrostatic attraction between positively charged peptide side chains and deprotonated silanol groups is a primary driver for adsorption.^19−21^ Another factor which can influence peptide adsorption is the hydrophilicity/hydrophobicity of the silica support itself, which can be induced by either post-grafting an alkyl silane onto the surface or co-condensation of an organic-bridge-containing bisilane. For both cases, it is known that the silica surface can turn very hydrophobic and may offer the possibility of attractive interactions with hydrophobic side-chain functionalities of the peptide.^15,22^

An attractive experimental setting for studying adsorption onto silica is to use a macromolecule adsorbate, like a peptide, that exhibits moieties of differing base strength, as the effective charge of the adsorbate then can easily be fine-tuned through pH changes. Literature data suggest that such molecules will likely show high loading capacities close to the peptide’s isoelectric point (pI), i.e., at low peptide/protein charge conditions, where the intermolecular electrostatic repulsion at the surface is low. It is known that peptide–peptide interactions play a pivotal role over peptide–surface interactions when it comes to the packing density reached.^23^ While it is often very intriguing to compare peptide and protein adsorption data with model isotherms like Langmuir, Freundlich, or Brunauer–Emmett–Teller (BET) models, Latour^24^ pointed out in an extensive study that most of the main assumptions underlying such models are not met for peptide/protein adsorption. He suggested that the points on the adsorption isotherm do not represent equilibrium states of surface coverage but rather concentration-dependent changes in areal density of the adsorbed peptide layer. Surely, under the consideration of curved surfaces and restricted space of a porous adsorbent, additional effects on the adsorption may arise.

A commonly used model introduced by Sang, Vinu, and Coppens (SVC) for estimating maximum peptide and protein uptake on porous adsorbents assumes that the entire pore volume can be ascribed to cylindrical pores with a uniform diameter into which peptide molecules can be fitted depending on the reduced pore diameter (ratio of pore diameter and peptide diameter).^25^ While the SVC model provides foundational understanding of the adsorption of proteins into mesopores, it yet falls short when applied to materials with nonuniform pore systems and shapes, as well as for proteins and peptides undergoing conformational transitions during adsorption. Meissner et al. enhanced this model by accounting for parts of the pore volume being inaccessible to the peptide, improving predictions for the experimental adsorption of lysozyme on SBA-15 materials by considering the secondary (micro)porosity of the material.^26^ A macroscopic effect of physically excluding adsorptive from interaction with the surface due to pore entry size constraints was demonstrated by Katiyar et al. by the adsorption of bovine serum albumin (BSA) onto SBA-15 with different pore sizes.^27^ They found that BSA adsorption was strongly depending on the pore diameter with very low loading for pore diameters <10 nm, but which increased by a factor of about 10 for 25 nm pores. It is expected that there might be pH-dependent alterations in the peptide’s behavior due to charge modulation, especially near its pI, which may include dimerization/aggregation and conformation-dependent changes in hydrophilicity.^8^ A confinement-related effect influencing the surface coverage of peptides and proteins is the increase in surface area the adsorbate occupies on convex-curved surfaces as compared to flat surfaces, as demonstrated by Yin et al. for the adsorption of an amyloid-β peptide onto graphene.^28^ Furthermore, Su et al. found that depending on the loading concentration and peptide charge, elliptical peptides like lysozyme tend to adsorb in different orientations, like head-on, side-on, or in a tilted orientation to achieve high surface coverage.^23^ These factors, beyond fundamental electrostatic or hydrophobic interactions, add a great deal of complexity to the adsorption on a plain silica surface and even more to the adsorption on curved surfaces within confined pore spaces. Our analysis aims to account for this multitude of factors, offering a nuanced understanding of peptide adsorption onto mesoporous silica. Unlike preceding studies predominantly focused on rigid proteins and a narrow range of MSNs, our research focuses specifically on peptides, employing theoretically modeled structures to elucidate adsorption variances across different pore systems.

In this study, we conducted pH-dependent adsorption trials using the cationic, low molecular weight peptide JM#21^29^ and a variety of MSNs characterized by differing pore size, geometry, and surface properties. Force field computations of the peptide were carried out in relation to the peptide charge at adsorption pH values to provide a theoretical framework for interpreting our findings. Particularly at pH levels near the peptide’s pI, which facilitate optimal peptide adsorption, we observed noteworthy variations in the peptide loading capacity across MSNs of different pore sizes. Comparing hydrophobic MSNs with their all-silica counterparts, we found enhanced adsorption onto hydrophobic MSNs under loading conditions devoid of electrostatic interactions, whereas under optimal adsorption conditions where electrostatic interactions prevail, the adsorption was diminished. To follow up, we conducted a functional cell assay for the peptide, effectively demonstrating its protection under serum conditions, i.e., environments where the peptide would typically exhibit a short half-life time. This understanding, integrated with the capability to predict loading based on peptide charge profile and potentially its conformation, unveils a pathway toward optimizing loading conditions and maximizing loading degrees. By aligning the suitable silica particle characteristics with the peptide attributes, this study lays a cornerstone for tailoring MSN systems, enhancing their potential for diverse therapeutic applications.

## Results and Discussion

### Physicochemical and Theoretically Modeled Properties of Peptide JM#21

**Figure 1:**
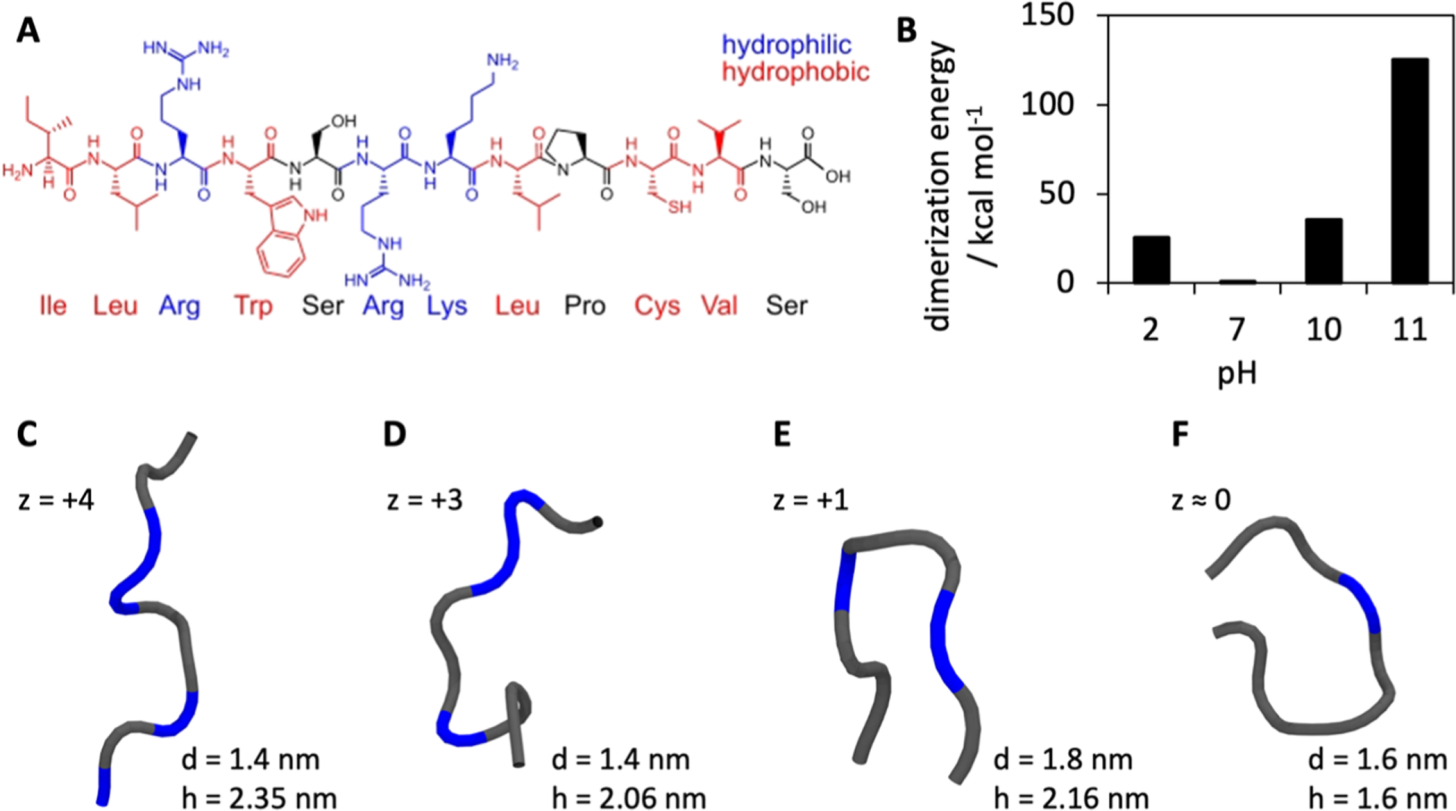
(A) Amino acid sequence of JM#21 with hydrophobic and hydrophilic amino acids indicated. (B) pH-dependent peptide dimerization energies calculated via molecular dynamics simulations. (C–F) Rendered structures of JM#21 obtained by molecular dynamics simulations in dependency of the peptide charge state/pH. The location of the positively charged amino acids giving rise to the net-charge z is indicated in blue.

JM#21 is a novel optimized derivative of the endogenous, peptide-based CXCR4 antagonist, EPI-X4, isolated from human hemofiltrate.^29^ While not being an antimicrobial peptide (AMP) itself, JM#21 is a good representative of typical AMPs from a chemical standpoint, enabling the transfer of the adsorption data of JM#21 onto silica to a whole class of widely applied therapeutic peptides. JM#21 shows rapid degradation in human plasma, resulting in a short half-life of 6 min. The peptide is composed of 12 amino acids (H_2_N-ILRWSRKLPCVS-COOH) of which six can be classified as hydrophobic and three as positively charged and hydrophilic at neutral pH (Figure 1A). The amino acid sequence results in a molecular mass of 1457 Da and a pI of 11.2 with a net charge of +3 at pH 7. Upon increasing the pH value, the overall peptide net charge gradually decreases to +1 at pH 10 and to a neutral state at a pH of around 11 (Figure S1A). The distribution of basic amino acids within the peptide sequence shows that at maximum charge, four positive charges are distributed among the first seven amino acids, while the remaining five carry no charge. Among the charge-carrying amino acids, the terminal alpha-NH_3_^+^ group typically has a p*K*_a_ value of around nine, and the side chains of lysine and arginine have p*K*_a_ values of 10.3 and 12, respectively. Consequently, upon reduction of the peptide net charge to +1, the charge distribution shifts toward the amino terminus with charges at amino acids three and six. Approaching the pI of the peptide, the remaining positive charge is either located at amino acid three or six, which in the case of three implies an even stronger shift of the positive charge toward the amino terminus. It is important to consider that these different charge states may not only be induced by the pH of the adsorption solution but also partial charge screening effects that occur upon interaction of the peptide with the negatively charged silica surface. Furthermore, we conducted theoretical calculations of the peptide’s 3D structure and its tendency to form aggregates at different charge states, which were representative for the pH values used for the adsorption study (Figure 1B). At a high charge (z = +4), the structure tends to be elongated (Figure 1C), while at a lower charge of +3, a loop structure is formed at the *C*-terminus (Figure 1D). At an effective charge of +1 and 0, the peptide transitions into a more compact conformation as a result of further loss of charged amino acid side chains, rendering the peptide more hydrophobic (Figure 1E,F). Furthermore, we examined these structures regarding their potential to dimerize and found that conditions close to the pI of the peptide energetically favor this process. The dimerization energies were calculated for the peptide under vacuum and should be lower by a factor of 4–12 for water, assuming the same conditions as for hydrogen bonds in peptides.^30^ Keeping this in mind, the dimerization energies at charges +4, +3, and +1 would be in the range of 4–14 room temperature (RT) in aqueous conditions (1 RT = 0.593 kcal mol^–1^), while the dimerization energy around the pI is substantially higher and should be in the range of 17–70 RT under aqueous conditions. To obtain at least a rough estimate of the adsorbate dimensions and its spatial demand on a surface (Figure 1C–F), the obtained peptide conformations were geometrically approximated by fitting them into a cylinder volume. By this simplification approach, we could get a general idea of realistic monolayer packing densities (PD_mono_) of minimum energy peptide structures at different charge states, neglecting interpeptide electrostatic effects. The charge distribution under different pH conditions suggests that the peptide will likely adsorb side-on (high charge, broadly distributed) or head-on (low charge, oriented toward amino terminus) onto the silica surface. Based on the derived cylinder dimensions, we calculated PD_mono_ for a head-on (hexagonal dense packing of cylinder base) and side-on (rectangular representation of cylinder side view) adsorption scenario (Table 2). In the head-on scenario, PD_mono_ is high at maximum charge due to the small cylinder diameter, reaches a minimum at z = +1, and increases again for the very compact peptide conformation near the pI. It is worth to note, though, that the very high PDs for head-on at high charge are probably unrealistic, as peptide–peptide repulsion should occur. The side-on scenario shows a similar trend with overall lower values for the PD, this time with a maximum at z = 0, a minimum at z = +1, and similar values for high charges. The maximum PD_mono_ of JM#21 consequently is expected to be in the range of 0.4–0.75 μmol m^–2^ when the influence of repulsive electrostatic or confinement effects on the adsorption can be neglected.

**Table 1:** Characterization of the MSNs^a^

	NSN	MSNhex	DMSN	DMON	DMONc
diameter/nm	206 ± 18	121 ± 12	158 ± 7	128 ± 11	128 ± 11
surface area/m^2^ g^–1^	23	1148	475	662	567
pore diameter/nm		3.5	4–12	3–20	3–20
pore volume (0.9 p p0–1)/cm^3^ g^–1^		0.82	0.51	0.89	0.91
zeta potential/mV	–58 ± 5	–35 ± 8	41 ± 8	–19 ± 3	–29 ± 4

a Specific surface areas were determined by BET analysis of nitrogen sorption measurements at 77 K. The particles’ pore diameter and pore volume were determined by NLDFT analysis for silica (equilibrium model) in the relative pressure range from 0 to 0.9. Zeta potentials were measured in aqueous hepes buffer (pH 7.2, 25 mM). The particle diameter was determined by TEM.

**Table 2:** Maximum Packing Densities (P*D*_max_) of JM#21 Adsorption onto a Nonporous NSN and Theoretical Packing Densities (PD_mono_) of a Monolayer Based on Modeled Peptide Dimensions for the Peptide Adsorbing Head-On (hdp of Cylinder Base) or Side-On (Cylinder Side, Rectangular Representation) at Different pH Values^a^

	pH 2	pH 7	pH 10	pH 11
peptide net charge	+4	+3	+1	0
c_eq_/mM	0.97 ± 0.01	1.07 ± 0.01	1.02 ± 0.02	1.02 ± 0.01
P*D*_max_/μmol m^–2^	0.14 ± 0.05	0.24 ± 0.1	0.66 ± 0.15	0.74 ± 0.1
PD_mono_ (head-on)/μmol m^–2^	0.98	0.98	0.59	0.75
PD_mono_ (side-on)/μmol m^–2^	0.5	0.58	0.43	0.65

a The BET surface area of the NSN was 23 m^2^ g^–1^, and the initial peptide loading concentration was 1.5 mM.

### Characterization of Silica Nanoparticles

To investigate the basic charge- and pH-dependent effects on the peptide packing density of JM#21 on a fully accessible silica surface, nonporous all-silica particles (NSNs) were synthesized via the Stöber method (Figure S2). The particles had a mean diameter of 169 nm, a specific surface area of 23 m^2^ g^–1^, and a zeta potential of −58 mV measured in 25 mM HEPES buffer at pH 7.2.

**Figure 2:**
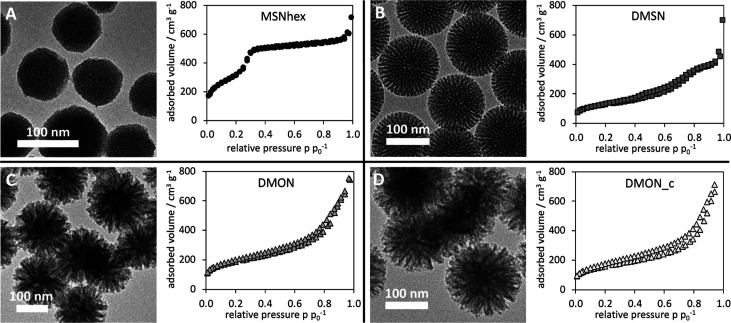
Transmission electron micrographs and nitrogen sorption measurements (conducted at 77 K) of the various mesoporous silica nanoparticles reveal clear structural differences of their pore system. A) MSN-hex, B) DMSN, C) DMON, and D) DMONc.

**Figure 3:**
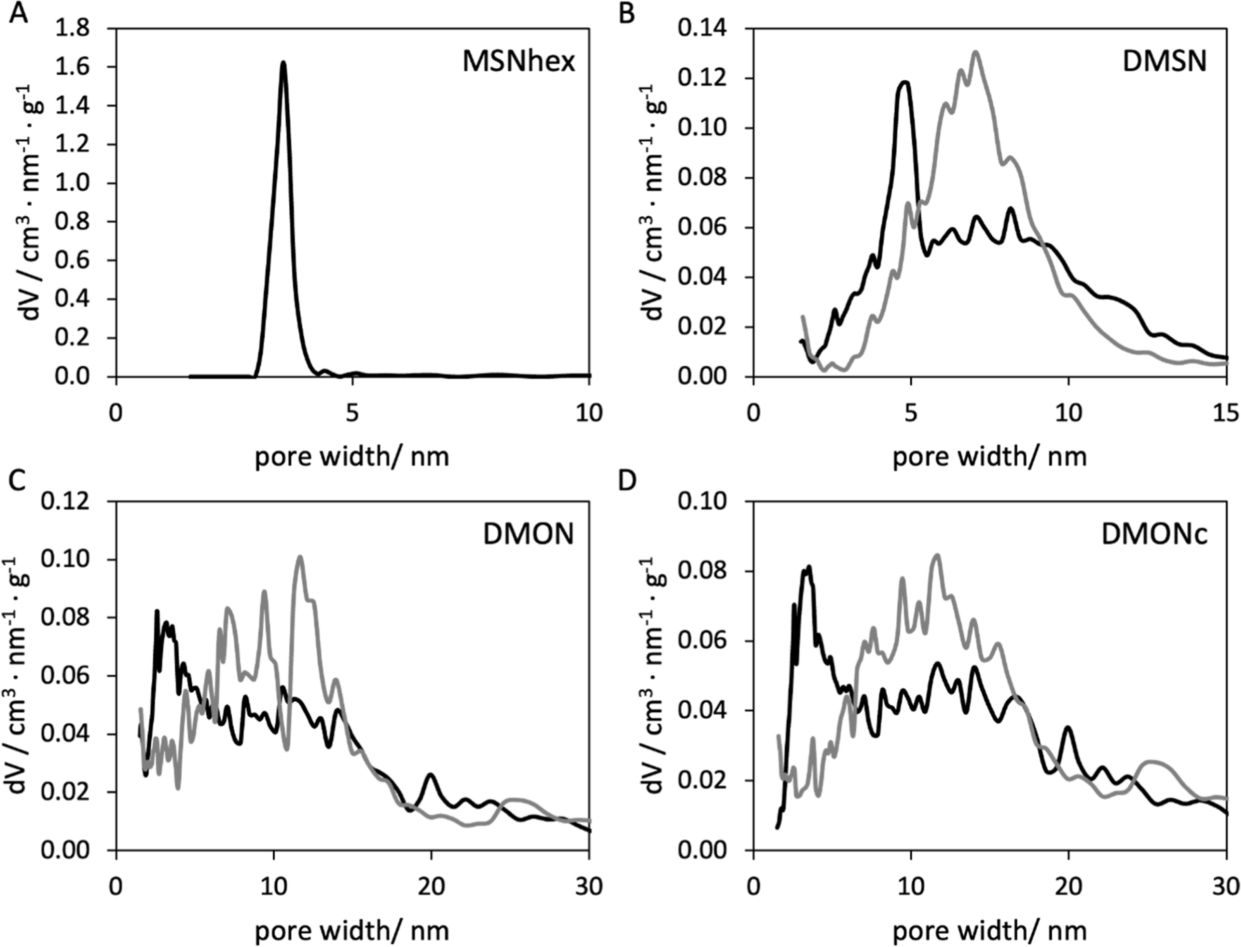
PSD curves of the different MSNs obtained by the NLDFT method from the equilibrium (black) and adsorption kernel (gray) of the nitrogen sorption measurements. (A) MSN-hex with a very narrow PSD of small pores and (B) DMSN, (C) DMON, and (D) DMONc with broad PSDs exhibiting maximum pore sizes of up to 12 (DMSN) and 20 nm (DMON/DMONc).

To evaluate the effect of different pore sizes and geometries on the adsorption of the peptide, a set of structurally different mesoporous all-silica nanoparticles were synthesized. MSNhex (Figure 2A) represent spherical nanoparticles with a hexagonally ordered system of cylindrical pores with relatively small pore diameters (3.5 nm) but a large specific surface area of 1148 m^2^ g^–1^ and a pore volume of 0.82 cm^3^ g^–1^. The hexagonal order of the pore system was confirmed by small-angle X-ray diffraction (SAXS) measurements (Figure S3) and fast forward Fourier transformation of transmission electron microscopy (TEM) images (Figure S4). The pore size distribution (PSD) was very narrow for MSNhex as can be seen in Figure 3A, evidencing the uniformity of the pore system. Dendritic mesoporous silica nanoparticles (DMSNs) also had a spherical shape but exhibited an inherently different pore structure than MSNhex. It can be described as a disordered system of nonuniform, roughly conical mesopores, which are radially arranged around the particle center. This could be confirmed by TEM images (Figure 2B) and the observation of only one broad reflex at low scattering vector values in SAXS measurements (Figure S3). The DMSN had a very broad PSD ranging from 4 to 12+ nm as shown in Figure 3B. The pore opening dimensions of the DMSN were estimated from TEM images and appear to be mostly represented by the higher range of the PSD around 8–12 nm. The pore diameter is gradually decreasing from the opening toward the particle center to around 3–4 nm, which is represented by the middle to lower diameter region of the PSD. The pore volume and specific surface area of the DMSN were smaller compared to the other types of MSNs with 0.51 cm^3^ g^–1^ and 474 m^2^ g^–1^, respectively.

The range of pore diameters of all-silica nanoparticles investigated was further extended by the synthesis of dendritic mesoporous organosilica nanoparticles (DMONs) and subsequent calcination, yielding their all-silica derivative DMONc, depicted in Figure 2C,D. The organic content of DMONs was determined to be about 20 wt % by thermogravimetric measurements (Figure S5), and the particles were structurally very similar to DMSNs (conical pores). In analogy to DMSNs, DMONs/DMONc exhibited a broad PSD, covering an even larger range of pore sizes (3–20+ nm) as can be seen in Figure 3C,D, with wide pore openings in the range of 10–20 nm, narrowing down to 3 nm toward the particle center. DMONs and DMONc had a specific surface area (662 and 567 m^2^ g^–1^) at a high pore volume (0.89 and 0.91 cm^3^ g^–1^), respectively. For all dendritic particles, the PSD was strongly dependent on whether the nonlocal density functional theory (NLDFT) analysis was performed based on the equilibrium or adsorption branch of the isotherm. Here, a consistent tendency was observed that the equilibrium kernel implied a higher fraction of smaller pores in the range of 3–5 nm (DMSN) and 3–10 nm (DMON/DMONc), while the adsorption kernel had its maximum at around 5–10 nm (DMSN) and 5–15 nm (DMON/DMONc). This could be indicative of partial restriction of larger pores by segments with a smaller diameter, in agreement with the overall irregular pore shape. Microporosity evaluation was done for all porous particles via the t-plot method. While no microporosity could be observed for MSNhex and DMSN, DMON and DMONc showed a small contribution of micropores, with a micropore volume of 0.067 and 0.051 cm^3^ g^–1^, respectively. A tabular summary of the physicochemical characteristics of the nanoparticles primarily covered in this study is given in Table 1. All particles had a negative zeta potential at neutral pH conditions, which was below −30 mV for the all-silica particles. DMON exhibited a higher zeta potential of −19 mV against −29 mV for DMONc, indicating a reduced surface silanol concentration resulting from the incorporation of hydrophobic moieties. Changes in zeta potential upon pH variation were investigated by measuring the zeta potential exemplary for all-silica MSNs in the loading buffers without peptide (Figure S6). As expected, the zeta potential was negative in the pH range of 7–11 but was close to neutral at pH 2 due to silanol protonation around the isoelectric point of amorphous silica (pH 2–3).^19^

### Peptide Packing Density on a Fully Available Surface Is Determined by Electrostatic Interactions

**Figure 4:**
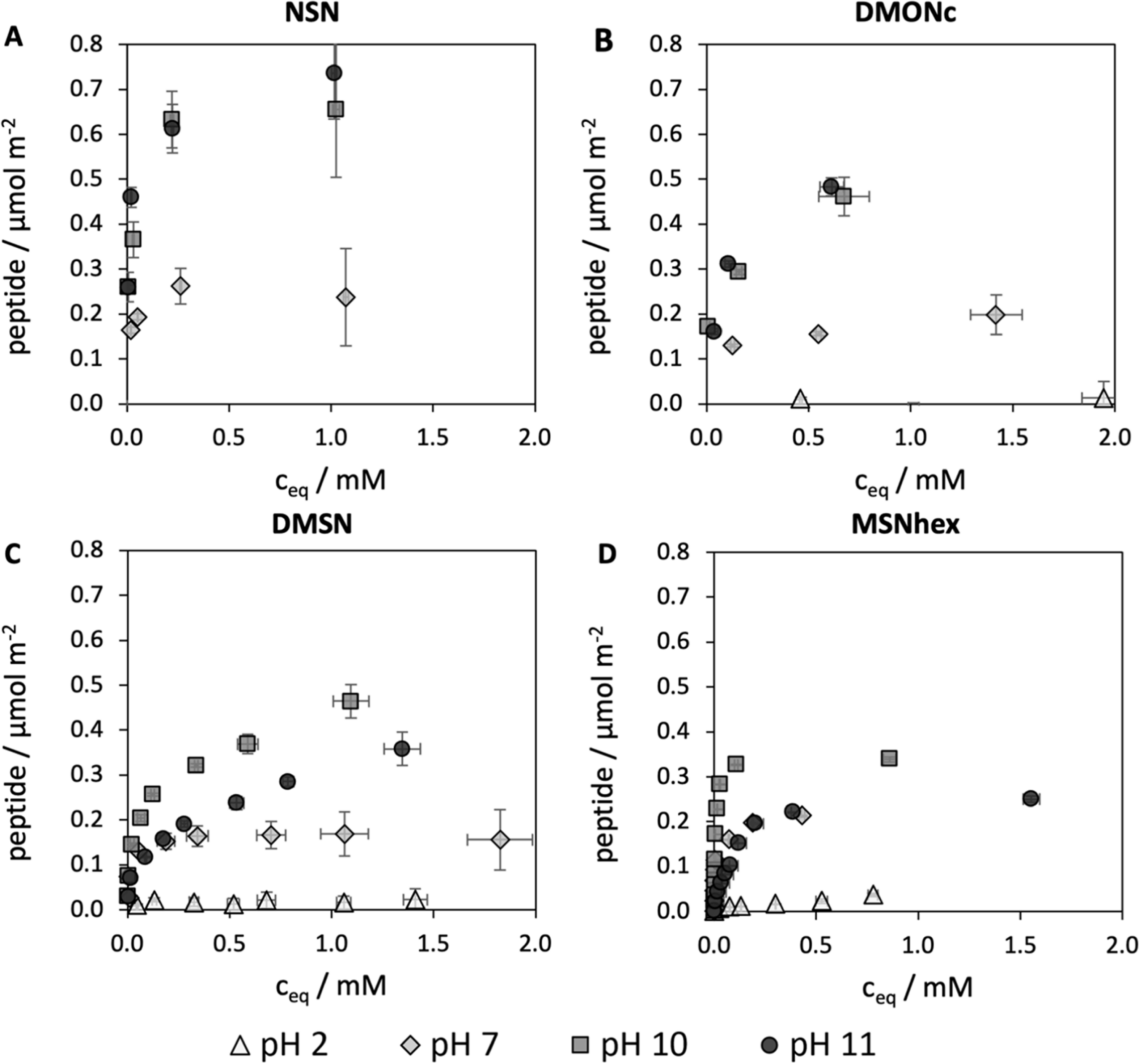
JM#21 adsorption isotherms onto (A) NSN, (B) DMONc, (C) DMSN, and (D) MSNhex recorded at different pH values. pH 2 (triangles), pH 7 (diamonds), pH 10 (squares), and pH 11 (circles) (*n* = 3, mean ± SD). Please note that no isotherm was included for the NSNs at pH 2 due to high data variance.

To elucidate the influence of peptide and silica charge on the PD on a relatively flat silica surface, adsorption isotherms of JM#21 onto nonporous silica nanoparticles (NSNs) at different pH values (2, 7, 10, and 11) were recorded, and peptide loading was normalized to the surface area to obtain packing densities. For the adsorption of a cationic peptide like JM#21 onto silica, electrostatic attractive forces between negative silanol groups and cationic side chains are reported to be a major contributor to the adsorptive interaction.^31^ For pH 2, it was not possible to get consistent data due to a combination of the low affinity of the peptide to silica and the very small surface area of the NSN. However, the results from the porous all-silica nanoparticles (Figure 4B–D) consistently showed that peptide adsorption was minor at pH 2. In contrast, the isotherm recorded at pH 7 was steep in the beginning before it gradually transitioned into a plateau around a PD of 0.28 μmol m^–2^ as can be seen in Figure 4A. The deprotonated silanol groups and highly charged peptide lead to strong electrostatic attraction and consequently to such a high-affinity isotherm. The maximum PD reached at the plateau is clearly lower than the calculated values from the modeled peptide dimensions, implying that the effective size of the peptide is much larger than that estimated by the model under these pH conditions. This effect can be explained by electrostatic repulsion between adsorbed peptide molecules increasing the effective size of JM#21 (repulsive charge field).^32^ Supporting a strong effect of charge on the effective surface occupancy, Su et al. found that lysozyme (pI of 11) occupies 14 nm^2^ at pH 8 (z = +8) versus 26.6 nm^2^ at pH 5 (z = +10).^23^ A similarly steep isotherm was observed at pH 10 but with a higher PD of 0.66 μmol m^−2^ reached at the plateau. The decreased peptide charge still allowed for strong attractive interactions with the silica surface, while peptide–peptide repulsion was significantly decreased, reducing the effective size of the peptide. The PD reached at this pH goes beyond the estimate for a side-on adsorption, ranging just around the calculated value for the head-on adsorption state. This is in alignment with the anisotropic charge distribution over the peptide structure at higher pH values, favoring head-on adsorption. A similar change in adsorption orientation was also observed by Su et al. in neutron scattering experiments.^23^ At pH 11, the isotherm was still steep at low equilibrium concentrations and transitioned toward a maximum packing density of 0.74 μmol m^–2^ with no distinct plateau reached under the investigated peptide loading concentrations. The PD again was clearly higher than the calculation for the side-on adsorption but fit well to values expected for head-on adsorption. The reduced affinity of the peptide toward silica may be balanced out by adsorption of dimers and aggregates (close to pI), leading to a relatively steep isotherm and high peptide PD due to possible lateral attractive interaction of the peptide adsorbing on the surface. The observations are in good agreement with literature reports stating that the adsorption of peptides is highly sensitive to the charge states of peptide and silica, respectively. As the surface of NSN is fully accessible for the peptide, the PD is mainly governed by charge-induced peptide properties, like effective size, conformation, and adsorption orientation. In all cases, the measured PDs either were below or equal to the calculated PDs based on peptide dimensions and preferential adsorption orientation, indicating that multilayer adsorption did not occur.

### Adsorbent Pore Size Limits the Peptide Adsorption onto MSNs

To evaluate the effect of different pore system characteristics on the peptide adsorption, adsorption isotherms of JM#21 onto MSNhex, DMSN, and DMONc (Figure 4B–D) were measured at the same pH values as for a NSN. The loading degrees derived by UV/vis measurements were exemplarily validated for JM#21-loaded MSNhex by thermogravimetric measurements (Figure S7).

As already stated above, at pH 2, all nanoparticle types exhibited similarly low peptide adsorption capacities. As virtually no peptide adsorbed at this pH, the contribution of van der Waals and hydrogen bonding forces to the adsorption can be assumed to be almost neglectable, irrespective of the pore size of the adsorbent.

In accordance with NSN, the adsorption isotherms at pH 7 showed a steep initial rise for MSNhex, DMSN, and DMONc, striving to a plateau for all particle types at a c_eq_ of around 0.19 mM. The PDs reached at the plateau were in a similar range for all porous particles (0.21 for MSNhex, 0.16 for DMSN, and 0.2 μmol m^–2^ for DMONc) and comparable, albeit slightly lower, to the values obtained for the NSN (0.24 μmol m^–2^). This leads to the conclusion that the maximum PD is independent of the pore size at pH 7 and mainly governed by the effective area that the peptide occupies on the surface. All adsorption isotherms at pH 7 showed a relatively flat transition from the initial steep rise into the plateau, which would be much more abrupt for, e.g., an ideal Langmuir isotherm with a similarly steep initial rise (high-affinity isotherm). This flat transition could be explained by effects like a change in the conformation or orientation of the adsorbed peptide, approaching maximum surface coverage under the given conditions as suggested by Latour.^24^ In the case of JM#21 at pH 7, the high charge state of the small peptide may lead to an initial adsorption in a flat conformation at low loading concentrations. This flat and spread-out conformation can be supported by the availability of multiple anchoring points, i.e., positively charged amino acid side chains. The calculated peptide structures show that JM#21 is very elongated at this pH, which is rather space demanding and, with increasing peptide concentration, may transform into a more compact conformation or tilted adsorption orientation, enabling higher surface PDs. In such a case, a strong binding of the peptide to the surface will lead to a pronounced flattening of the isotherm’s transition point into the saturation plateau.

The overall similar adsorption behavior of the three porous particles drastically changed at pH 10, where clear differences were observed in dependency of the pore characteristics. JM#21 having a lower but still net positive charge resulted in a steep initial rise of the isotherms for all particles, which could be observed up to a PD of 0.3 μmol m^–2^ for MSNhex and up to 0.15 and 0.19 μmol m^–2^ for DMSN and DMONc, respectively. These relatively small surface access values for DMSN and DMONc may indicate that part of the surface is inaccessible for peptide adsorption, as some microporosity was observed for DMONc, and both particles exhibit an irregular pore structure. Due to a smaller effective size of the peptide at this pH, the isotherm of MSNhex transitioned into a plateau at a higher maximum PD of around 0.34 μmol m^–2^ as compared to pH 7, which is still clearly lower than the respective value observed for NSN. The PD reached is even below the theoretical PD calculated for side-on adsorption at this pH, indicating that the small pore size clearly limits the dense packing of the peptide (confinement effect). In contrast to that, the less steep isotherms obtained for DMSN and DMONc transitioned into a linear upward progression at around 0.2 μmol m^–2^ with a measured maximum PD of 0.47 and 0.46 μmol m^–2^, respectively. The fact that no distinct adsorption plateau was observed indicates that with the larger pores, the peptide can approach a densely packed monolayer as there is enough space for PD-optimizing rearrangement processes. Consequently, the linear progression could be interpreted as a heavily extended transition phase into a plateau beyond the experimental conditions. The PD values were in the range of the theoretical values for side-on and below head-on adsorption, which shows that confinement limitations of the adsorption can also be observed at larger pore sizes. This is probably connected to the conical shape of the pores with decreasing pore diameters toward the particle center causing PD limiting confinement effects, as observed for MSNhex. These confinement effects should be mainly connected to the curvature of the adsorbent surface and the limited space provided by the pores. The surface curvature effect suggests a higher surface occupancy of an adsorbate molecule interacting with a negatively curved surface as described by Yin et al.^28^ The limited pore space not only physically restricts the extent of head-on adsorption in a densely packed monolayer but also has a negative influence on the ability of the peptide to rearrange into a conformation or orientation that is favorable for denser packing. At pH 10, the transition of the MSNhex isotherm into the plateau is less flat as compared to pH 7. This could indicate that at pH 10, the peptide either undergoes only a minor conformational transition (less flat initial adsorption) with increasing loading concentration or that the conformational transition comes at a lower energy cost as the peptide has less excess charge enabling for multipoint interactions with the surface. The reduced electrostatic repulsion should further lower the barrier for peptide rearrangement and dense packing on the surface.

At pH 11, the reduced attractive interactions with silica and an increased tendency to dimerize or even aggregate result in pronounced pore size-related effects on the adsorption. Signs of this lower attraction could be seen in the isotherms of MSNhex and DMSN, both of which revealed a lower affinity of the peptide to silica, resulting in a relatively flat isotherm compared to pH 7 or 10. For DMONc, however, the isotherm progressed in a steeper fashion up to a PD of around 0.18 μmol m^–2^ even at this pH. For the small pore MSNhex, the isotherm flattened out toward a maximum loading degree of around 0.25 μmol m^–2^ which is comparable to the value reached at pH 7, while the isotherms of DMSN and DMONc again transitioned into a linear continuation up to a PD of 0.36 and 0.48 μmol m^–2^, respectively. All particles reached lower maximum PDs within the investigated peptide concentration range compared to those of the NSNs (0.74 μmol m^–2^), which were even below the theoretical maximum PD for side-on adsorption. Interestingly, MSNhex and DMSN even reach a lower maximum PD as compared to pH 10, which is clearly different from what was observed for the NSN where the PD at pH 11 was even slightly higher than at pH 10. This can be explained by a combination of pronounced confinement effects within the pores (lateral stacking of the peptide in a dense layer) and pore size-dependent inhibition (size exclusion) of the adsorption of peptide dimers and potential larger aggregates. It is suggested that the larger pore openings of DMSN provide at least some accessibility of the surface to peptide aggregates and sufficient space for conformational or orientational changes, causing the linear continuation of the isotherm. In contrast to that, the small pores of MSNhex do not provide enough space for these processes, leading to a pore size-limited plateau already at lower packing densities. Similar observations of loading capacity limiting confinement in dependency on the pore size were made by Andersson et al.^33^ for the adsorption of Ibuprofen on MSN with different pore sizes, as well as by Katiyar et al.^27^ adsorbing BSA onto SBA-15. For DMONc, however, the loading degree reached at pH 11 is similar to pH 10, and the isotherm shapes were almost identical for both pHs, as observed for NSN. This suggests that the very large pores of DMONc provide higher accessibility of the surface to peptide dimers and aggregates, however not to the same extent as the fully available surface of the NSN, which can be related to the large pores becoming narrower toward the particle center. This adsorption of peptide dimers/aggregates was also reflected in the steeper rise of the isotherm of DMONc.

To check for pore network effects aside of pore shape and PSD, the peptide adsorption onto MSNhex, MSNrad, and MSNrod which have a very similar pore diameter, but different pore lengths and connectivity, was investigated. MSNrad and MSNrod had similar specific surface areas (986 and 1031 m^2^ g^–1^) as well as pore diameters (3.2 and 3.7 nm) as MSNhex, respectively. The physicochemical properties of the particles are summarized in Table S1. TEM images, nitrogen sorption isotherms, and PSDs are shown in Figure S8. In contrast to MSNhex, MSNrad had pores that were radially aligned around the particle center, making the pores only available through one opening. Elongated MSNrod particles (aspect ratio (AR) = 2) exhibited pores aligned along the longitudinal axis, resulting in double the pore length of MSNhex (Figure S9). Across the range of pH values investigated, all the three particle types reached a comparable peptide loading, as shown in Figure S10 (MSNrod at pH 2 not shown). This result shows that a comparable amount of the pore surface area is accessible for the peptide, irrespective of the pore length and connectivity. Consequently, the diffusion length of the peptide must be long enough to not become a limiting factor for the adsorption and does not affect the results discussed for MSNhex, DMSN, and DMONc.^34^

### Hydrophobic Interactions Facilitate the Adsorption of JM#21 under Electrostatically Unfavorable Conditions

To investigate the possibility of promoting peptide adsorption by enabling additional hydrophobic interactions, adsorption onto DMON, containing a benzene-bridged silica network, was investigated. The benzene bridges provide not only classical hydrophobic interactions in the form of van der Waals forces with hydrophobic amino acid side chains but also π–π interactions (quadrupolar interactions) with other aromatic moieties, like the aromatic functionality of tryptophane. The hydrophobic functionalization via co-condensation was chosen over post-functionalization, as the latter technique is known for having a negative influence on the PSD of the particles, making them not comparable to the all-silica reference. As already highlighted in the nanoparticle characterization, the removal of the organic moieties via calcination preserved the structure of DMON, making the resulting DMONc a perfect all-silica reference for these experiments.

**Figure 5:**
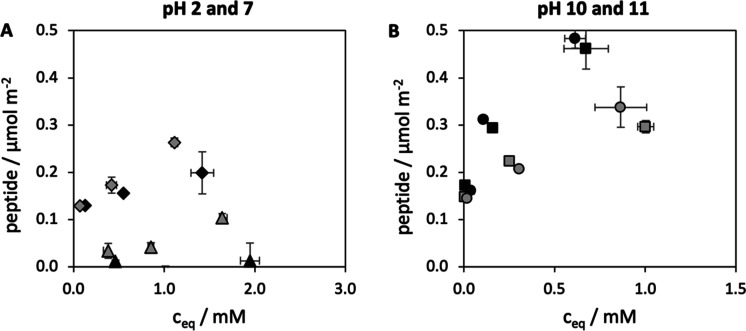
JM#21 adsorption isotherms onto DMON (light gray) and their all-silica structural derivative DMONc (black) recorded at (A) pH 2 (triangles) and 7 (diamonds) and (B) pH 10 (squares) and 11 (circles) (*n* = 3, mean ± SD).

Adsorption of JM#21 onto DMON and DMONc was tested at different pH values for three different peptide loading concentrations, as shown in Figure 5. At pH 2, the adsorption onto calcined DMONc was negligible as discussed in the previous section (0.01 μmol m^–2^), whereas the PD on DMON was increased by a striking factor of 10 (0.1 μmol m^–2^). A similar but lower beneficial effect on the adsorption of JM#21 was observed at pH 7, where the DMON reached 0.26 μmol m^–2^ compared to 0.20 μmol m^–2^ for DMONc, i.e., values almost equal to the PD reached for the NSNs. It is known from the literature that silanol dissociation takes place over a broad range of pH, and a certain amount of the silanol groups could still be protonated at pH 7.^20^ This reduces electrostatic attraction as well as repulsive charge neutralization of adsorbed peptides, and therefore, a positive contribution of hydrophobic interactions can still be plausible at this pH. In contrast, at pH 10 and 11, peptide loadings were inversed, and the DMONc outperformed the DMON, reaching about 50% higher PD. These results show that under unfavorable charge conditions, the presence of hydrophobic moieties within the silica network promotes adsorption by enabling hydrophobic interactions, while this effect diminishes under pH conditions, where the electrostatic interactions dominate adsorption. In the latter case, the lower surface concentration of silanol groups on the organo-functionalized DMON, which was indicated by the lower zeta potential of DMON (−19 mV) compared to DMONc (−29 mV), led to a decreased peptide adsorption capacity as compared to the all-silica particles. The fact that at pH 11, where electrostatic contributions to the attraction were reduced, hydrophobic interactions did not positively contribute to the adsorption (other than at pH 2 and 7) demonstrates the dominance of hydrophilic interactions still present at this pH. Interestingly, the adsorption isotherms on these large-pore particles generally do not seem to approach a plateau under the investigated adsorption conditions, and even the isotherms for pH 2 and 7 show no obvious signs of approaching a plateau, especially for DMON.

### Adsorption onto MSN Retains Peptide Activity Independent of the Pore Size

**Figure 6:**
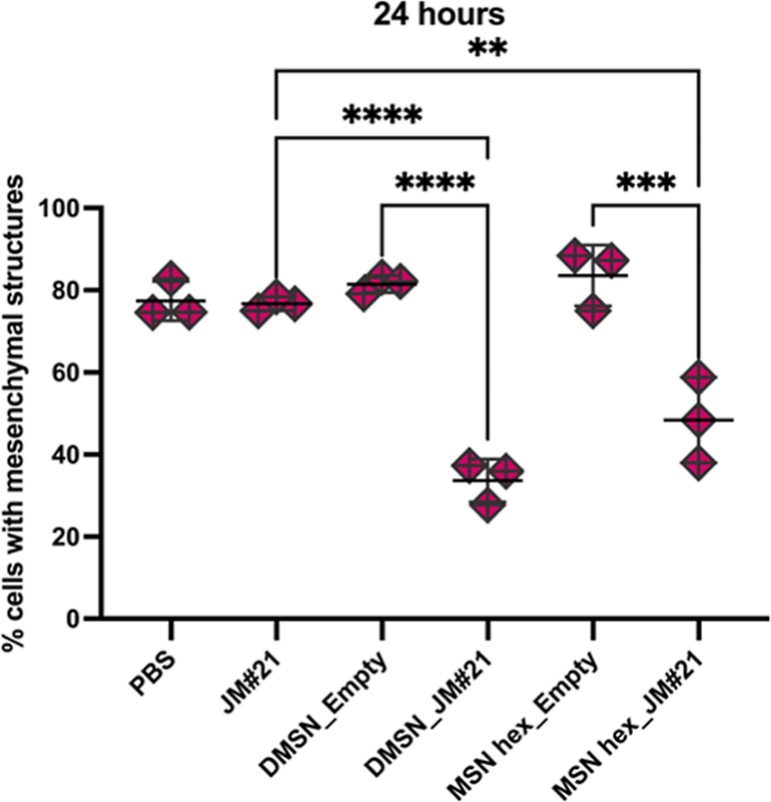
CXCL12-induced changes in the actin structure of Panc354 cells treated with CXCL12 (10 μM), together with PBS, JM#21 (10 μM), nonloaded MSNhex and DMSN (empty), or peptide-loaded MSNhex (30 wt % JM#21) and DMSN (27 wt % JM#21) in RPMI/10% FCS for 24 h at 37 °C. The particle concentration was normalized to 10 μM JM#21 at full release (*n* = 3, mean ± SD, *p ≤ 0.05, two-way ANOVA).

Zeta potential measurements of MSNhex loaded with different amounts of peptide revealed only minor shifts toward less negative values, indicating that the peptide should reside mainly inside the pore system and provide a shielding effect against enzymatic degradation (Figure S11). To investigate pore size effects on the shielding of the peptide and to prove the conserved biological activity, a cell-based *in vitro* functional assay was performed. This assay is based on the reversible binding of the chemokine CXCL12 to the CXCR4 receptor of the primary pancreatic cancer cell line, Panc354. Upon internalization of CXCL12, remodeling of the cytoskeleton toward a mesenchymal-like form is induced. However, this structural change can be suppressed by blocking CXCR4 via irreversible binding with JM#21. Thus, inhibition of cytoskeletal changes can be used as a measure of peptide functionality and analyzed via fluorescence microscopy (Figure S12). Incubation of Panc354 cells with either free JM#21 or JM#21 loaded onto MSNhex and DMSN enabled monitoring of the biological effect of the released peptide in the presence of serum proteins, i.e., under conditions where the free peptide is rapidly degraded and nonfunctional. MSNhex and DMSN were loaded under optimal loading conditions (pH 10) and had a high loading degree of 30 and 27 wt %, respectively. The concentration of loaded nanoparticles was chosen to match the applied concentration of free JM#21 in the case of full release from the nanoparticles. Figure 6 shows the relative number of Panc354 cells with morphological changes to a more prominent mesenchymal phenotype as compared to the total number of cells after treatment for 24 h. The cells exposed to the free peptide showed CXCL12-induced structural changes to the same extent as the PBS control (CXCL12 only). JM#21 is reported to be readily degraded in human serum resulting in a short half-life time of 6 min.^29^ Proteolytic degradation also happened in this case, as the assay was performed in serum containing cell culture medium. However, incubation with DMSN or MSNhex loaded with JM#21 clearly reduced the extent of mesenchymal actin structures by a factor of about 2, whereas the unloaded nanoparticles had no influence on the cells. It is important to note that the particles had no cytotoxic effects on the cells (Figure S13). These results not only demonstrate the release of JM#21 in a functional form and the preservation of its ability to bind to CXCR4 but also display effective peptide protection against serum protein-mediated degradation by MSNs. A similar activity level of JM#21-loaded MSNhex and DMSN shows that even the large pores of DMSNs provided efficient shielding of the peptide against degradation.

## Conclusions

In this study, we investigated the effect of several characteristics of MSNs like pore size and structure as well as surface charge and hydrophobicity on the adsorption of the short, cationic peptide JM#21 under various pH conditions. With variation of the pH, the charge of the peptide could be modulated, resulting in changes of electrostatic interactions, peptide conformation, and aggregation, which had striking effects on the adsorption behavior of the peptide. By modeling of the peptide structure at different charge states, the monolayer PD on a surface could be estimated to be in the range of 0.4–0.75 μmol m^–2^. Adsorption studies of JM#21 onto NSNs providing a fully accessible silica surface showed that the PD is highly sensitive to the electrostatic interactions between adsorbate molecules as well as between adsorbent and adsorptive. The highest PDs were reached near the pI of the peptide and were comparable to the maximum theoretical PD values. Adsorption was reduced under conditions in which the strong peptide–silica interaction was countered by peptide–peptide electrostatic repulsion (pH 7) or completely diminished under conditions in which the silica surface was uncharged. Importantly by comparing experimental PDs with the theoretical PD_mono_ values, multilayer adsorption could be excluded, which is an important prerequisite for the discussion of the adsorption data on MSNs.

While confirming the established trends based on electrostatic interactions, adsorption of JM#21 onto MSNs with different pore sizes and therefore a surface area with variably restricted access revealed a multitude of confinement-related effects on the adsorption. Especially under optimal loading conditions (pH 10 and 11), we discovered a strong dependency of the PD on the pore sizes of the particles. Peptide adsorption onto the MSNs was likely influenced by the confinement effects, restricting the peptide’s space to achieve packing in a dense monolayer of favorable orientation and conformation. Additional factors include pore size restriction of the peptide dimer and aggregate adsorption and surface curvature effects that increase the effective surface covered by the peptide in the adsorbed state. By combination of the data on peptide charge and conformation with detailed structural information about the pore system of the different particles, a comprehensive understanding of the experimental isotherms could be gained.

The influence of surface hydrophobicity on the peptide adsorption was investigated by loading JM#21 onto DMONs that contain benzene-bridged silanes within the silica network. Particularly under unfavorable loading conditions (pH 2), where electrostatic interactions are screened, higher packing densities were reached for the hydrophobic DMON compared to their all-silica counterparts (DMONc). This effect was still observed at pH 7 but diminished at pH 10 and 11, where electrostatic attraction is the dominant driving force for the adsorption.

Moreover, in vitro studies showed that CXCL-12-induced morphological changes of Panc356 cells were diminished upon incubation with JM#21-loaded particles, contrary to the rapidly degraded free JM#21, proving the protective benefits offered by MSNs (even with large pores) for easily degradable peptide cargos.

## Experimental Section

### Chemicals and Materials

Tetramethyl orthosilicate (TMOS), (3-aminopropyl)trimethoxysilane (APTMS), tetraethyl orthosilicate (TEOS), 1,4-bis(triethoxysilyl)-benzene (BTEB), cetyltrimethylammonium chloride solution (CTAC) (25 wt % in water), triethanolamine (TEA), Phalloidin-Atto565, and paraformaldehyde (PFA) were purchased from Sigma-Aldrich Chemie GmbH, Schnelldorf, Germany. Cetyltrimethylammonium bromide (CTAB) and ammonium hydroxide (28 and 32% in water), sodium hydroxide, ethanol, methanol, acetone, ethylene glycol, sodium carbonate, sodium hydrogen carbonate, sodium dihydrogen phosphate, disodium hydrogen phosphate, cyclohexane, phosphoric acid, and hydrochloric acid were purchased from VWR International GmbH, Darmstadt, Germany. Ammonium nitrate was purchased from Carl Roth GmbH & Co. KG, Karlsruhe, Germany. Phosphate-buffered saline (PBS) was purchased from Thermo Fisher Scientific, Karlsruhe, Germany.

The CXCR-4 antagonist, JM#21, was kindly provided by Mirja Harms and Jan Münch (Institute for Molecular Virology, Ulm). The peptide was used without further purification. Six-well plates were purchased from Greiner Bio-One, Frickenhausen, Germany. FBS, RPMI (Gibco), glutamine (Gibco), and Prolong Gold reagent with DAPI were purchased from Thermo Fisher Scientific, Waltham, USA. Penicillin–streptomycin was purchased from PAN Biosystems, Aidenbach, Germany. CXCL12 was purchased from PeproTech, Cranbury, USA. Sodium salicylate (NaSal) and TritonX-100 were purchased from Fluka, Charlotte, USA.

### Synthesis of DMSNs

DMSNs were synthesized according to a protocol in the literature.^35^ TEA (0.45 g, 3.025 mmol) was dissolved in a CTAC solution (25 wt % in water, 60 mL, 45.375 mmol CTAC) and ultrapure water (90 mL). The solution was stirred for 30 min at 60 °C. The water phase was then overlaid with a solution of TEOS (10 mL, 44.775 mmol) in cyclohexane (40 mL). The reaction mixture was kept at 60 °C and stirred at 100 rpm for 48 h. The DMSNs were isolated by separating the water phase, diluting it with ethanol (90 mL), and centrifugation (15,650 rcf, 30 min). After washing twice with ethanol, the DMSNs were dried at 60 °C, ground to a fine powder, and calcined at 550 °C for 5.5 h (1.83 K min^–^1 heat ramp).

### Synthesis of DMON and DMONc

DMONs with a benzene-bridged silane were synthesized according to a protocol based on Kalantari et al.^36^ CTAB (380 mg, 1.04 mmol), NaSal (84 mg, 0.52 mmol), and TEA (68 mg, 0.45 mmol) were dissolved in ultrapure water (24 mL) and equilibrated at 80 °C for 60 min. Then, TEOS (2.68 mL, 12 mmol) was added, followed by BTEB (2.34 mL, 5.93 mmol) with a delay of 15 min. The reaction was stirred at 450 rpm at 80 °C for 18 h before the DMONs were isolated by diluting the cooled reaction mixture with ethanol (50 mL) and centrifugation (15,650 rcf, 12 min). The surfactant (CTAB) was removed by extraction with acidified ethanol (4 g of HCl per L of ethanol) for 1 h in an ultrasonic bath (repeated three times). After that, the DMONs were dried at 60 °C and ground to a fine powder.

The calcined, all-silica derivative of DMON (DMONc) was obtained by calcination of the DMON at 550 °C for 5.5 h (1.83 K min^–1^ heat ramp).

### Synthesis of MSNrad

MSNs with a radially aligned pore system (MSNrad) were synthesized according to Rosenholm et al.^37^ After dissolving porogen CTAB (7.88 g, 21.6 mmol) in a mixture of methanol (860 mL), water (909 mL), and sodium hydroxide solution (1 M, 4.56 mL, 4.6 mmol) in a 2 L round-bottomed flask, the mixture was equilibrated to RT. A mixture of silica precursors TMOS (2.18 mL, 14.7 mmol) and APTMS (0.36 mL, 2.1 mmol) was added at a stirring rate of 500 rpm. The stirring rate was adjusted to 300 rpm after 45 min, and the reaction proceeded for 16 h. Upon precipitation with ammonium nitrate (35 g, 27.4 mmol), the particles were separated via centrifugation and subsequently washed once with water and twice with ethanol in an ultrasonic bath for 1 h each. Finally, the particles were dried at 60 °C in vacuo and calcined at 550 °C for 5.5 h (heating rate 1.8 °C min^–1^) yielding all-silica “MSNrad”.

### Synthesis of Nonporous Stöber Silica Nanoparticles

Solid, nonporous silica nanoparticles were synthesized as reported elsewhere.^38,39^ The silica precursor, TEOS (3 mL, 13.5 mmol), was added to a mixture of ethanol (55.8 mL), water (44 mL), and ammonium hydroxide solution (28 wt %, 14.8 M, 6 mL, 100.7 mmol) in a 0.5 L round-bottomed flask. The mixture was stirred at a rate of 450 rpm for 19 h at RT. The particles were separated via centrifugation, washed twice with ethanol in an ultrasonic bath for 1 h, and dried at 60 °C in vacuo. Finally, the particles were calcined as described above to yield all-silica “NSN”.

### Synthesis of MSNs with Varying AR (MSNhex and MSNrod)

MSNs with a hexagonal parallel-aligned pore system were synthesized based on the synthesis by Huang et al.^6^ CTAB (1.1 g, 3 mmol) was dissolved in a mixture of water (270 mL) and ammonium hydroxide solution (32 wt %, 16.6 M, 5.7 mL, 94 mmol) in a 0.5 L round-bottomed flask. To obtain particles with a lower AR, ethylene glycol (110 mL, 1.62 mol) was added. Upon addition of TEOS (4.7 mL, 21.2 mmol), the mixture was stirred with 600 rpm for 4 h. The particles were precipitated with ammonium nitrate (5 g, 3.9 mmol), separated via centrifugation, washed twice with ethanol in an ultrasonic bath for 1 h, and dried at 60 °C in vacuo. Finally, the particles were calcined as described above for 8 h to yield all-silica “MSNhex” (AR = 1.1) and “MSNrod” (AR = 2).

### Characterization of Silica Nanoparticles

The particles’ diameters and pore structures were examined with a Jeol 1200 (Jeol, Germany) transmission electron microscope using a HT voltage of 120 kV and a beam current of 65 μA. Fast Fourier transformation (FFT) was used to obtain grayscale images of the electron diffraction patterns using the FFT plug-in of ImageJ version 1.52n. By filtering different frequency ranges via selection of gray values ranging from 158 to 178 or from 146 to 160 and subsequent inverse FFT calculations, electron diffracting structures were visualized. Small-angle X-ray scattering (SAXS) was performed on a Bruker Nanostar (Bruker, USA) using Cu Kα radiation (λ = 1.5406 Å) at an angle from 0.3 to 5° (2θ). Nitrogen sorption measurements were conducted at −196 °C on a Quadrasorb-1 (Quantachrome Instruments, Germany) after drying the particles in vacuo at 100 °C for 22 h. The pore diameters were calculated via the equilibrium NLDFT kernel (silica, cylindrical pores) in the relative pressure range of 0–0.9, and the pore volumes were determined at a relative pressure of 0.9. The specific surface areas were determined by using the BET method. By using the t-plot method in the relative pressure range of 0.75–0.95, the external surface areas were estimated. Zeta potentials were measured with a Zetasizer NanoZS Zen3600 (Malvern Panalytical, Germany) at a particle concentration of 0.1 mg mL^–1^ in the aqueous buffer solution as stated. Thermogravimetric analysis was performed at a heating rate of 10 °C min^–1^ in a nitrogen/oxygen (70%/30%) atmosphere on a TG209 F1 Libra (NETZSCH, Germany).

### Peptide Adsorption onto Silica Nanoparticles

JM#21 adsorption was conducted in different aqueous buffer solutions: 12 mM carbonate buffer (pH 10 and 11) and 12 mM phosphate buffer (pH 2.1 and 7.4). Stock solutions of JM#21 (5 mM) in each buffer were prepared, and the pH was adjusted with sodium hydroxide solution (1 M). The different particle types were dispersed in each buffer by using a focused ultrasonic bath. After adjusting the pH value with sodium hydroxide solution, particle dispersion, peptide stock, and buffer were mixed in 2 mL centrifugal tubes (Eppendorf), resulting in a constant particle concentration of 5 mg mL^–1^ and varying peptide concentrations (0–4 mM). Upon rotating the mixture for 2 h at RT, the particles were centrifuged (14,800 rpm; 5 min) and briefly washed with acetone. The particles were dried in vacuo at 60 °C. The amount of peptide adsorbed onto the particles was determined by measuring the absorbance of the adsorption supernatants at 280 nm by using UV/vis spectroscopy.

### Peptide Modeling

Molecular dynamics simulations were used to investigate the self-interactions of the JM#21 peptide. For this purpose, the atomic positions of the peptide structures with different pH values were used and equilibrated at 300 K through ReaxFF simulations in the Amsterdam Modeling Suite 2021 (http://www.scm.com) for 0.5 ns. Subsequently, two peptides were placed in the simulation box, and the system was equilibrated again for 0.5 ns at 300 K using ReaxFF simulations. Finally, the interaction energy was determined and averaged by running ReaxFF simulations in the *NVT*-ensemble for 25 ps while the system was coupled to a Berendsen heat bath with a temperature of 300 K and a coupling constant of 100 fs.

### Cell Culture and Activity Assay

Primary human pancreatic cell lines (Panc354) were generated from the resected excess pancreatic carcinoma tissue that was subcutaneously implanted into nude mice in vivo.^40^ Panc354 cells were maintained at 37 °C in a humidified atmosphere with 5% CO_2_ in culture medium RPMI supplemented with 10% FBS, 1% penicillin–streptomycin, and 1% glutamine. For experimental setup, cells were used in early passages (Pass. 3–20) and were recovered from frozen stocks on a regular basis. Single cells were counted with Neubauer chambers and seeded accordingly.

Panc354 cells were cultured at a density of 50,000 on coverslips (24 × 60 mm, Menzel-Gläser) in a six-well plate for 24 h until attachment. Afterward, the cells were treated with CXCL12 (10 μM), together with PBS, JM#21 (10 μM), nonloaded MSNhex and DMSN, or peptide-loaded MSNhex (30 wt % JM#21) and DMSN (27 wt % JM#21). The final silica concentrations of MSNhex and DMSN were 34 μg mL^–1^ and 37 μg mL^–1^, respectively, which corresponded to 10 μM peptide, assuming full release from the loaded particles. After treatment for 24 h, the cells were washed three times with PBS. Subsequently, they were fixed with 2% PFA for 20 min at RT and then washed again three times with PBS. Next, the cells were permeabilized with 0.7% TritonX-100 solution for 15 min at RT, followed by three PBS washing steps. Coverslips with cells were then incubated with Phalloidin-Atto565 (1:500 diluted in PBS) inside a dark humid chamber for 60 min. Finally, the cells were washed three times in PBS and mounted on slides using Prolong Gold reagent with DAPI. Fluorescence microscopy was performed at 353 nm (cell nuclei) and 565 nm (cytoskeleton) using a Zeiss Axio Vert.A1 with an EC Plan-Neofluar 63*x*/1.25 oil objective and ZenBlue software. The experiments were conducted in triplicate (*n* = 3), and five representative pictures were taken per experiment and processed with ImageJ. The mesenchymal cytoskeletal remodeling was quantified by counting cells with stress fibers, spindle-like, and elongated actin structures in relation to the total number of cells per picture.

## Data Availability

The reported data are either mean values ±SD or individual values. The sample size (*n*) is specified in the respective figure legends and table captions. Statistical significance was tested with two-way ANOVA using GraphPad Prism.

## Supporting Information Available

Calculated peptide charge as a function of pH; NSN characterization results; SAXS measurements for the studied particles; TEM image and reconstruction of MSNhex particles; TGA results for DMONs and DMONc particles; pH-dependent zeta potential values for MSNrad particles; TGA results measured for JM#21-loaded MSNhex particles; characterization results for MSNrad and MSNrod particles; comparison of maximum JM#21 loadings into MSNrad, MSNhex, and MSNrod particles; zeta potential measurements of JM#21-loaded MSNhex particles as a function of peptide loading; and fluorescence microscopy images of CXCL12-treated Panc354 cells exposed to JM#21 and corresponding cell viability data (PDF)
