# Supplementary material for: Confinement and Polarity Effects on the Peptide Packing Density on Mesoporous Silica Nanoparticles
Source: Langmuir. 2024 Feb 12;40(8):4294–305. doi: 10.1021/acs.langmuir.3c03513 (PMC10905996; doi:10.1021/acs.langmuir.3c03513)
Supplement: Supplementary file 1 — la3c03513_si_001.pdf [file la3c03513_si_001.pdf]

Supporting Information

Confinement and polarity effects on the peptide packing density on mesoporous silica nanoparticles

Bastian Beitzinger,[1,#] Roman Schmid,[1,#] Christoph Jung,[2] Kanishka Tiwary,[3] Patrick Hermann,[3] Timo Jacob,[2] and Mika Lindén[1,*]

[1] Institute of Inorganic Chemistry II, Ulm University, Albert-Einstein-Allee 11, 89081 Ulm, Germany

[2] Institute of Electrochemistry, Ulm University, Albert-Einstein-Allee 47, 89081 Ulm, Germany

[3] Department of Internal Medicine I, Albert-Einstein-Allee 23, Ulm University, 89070 Ulm, Germany

[#] These authors contributed equally

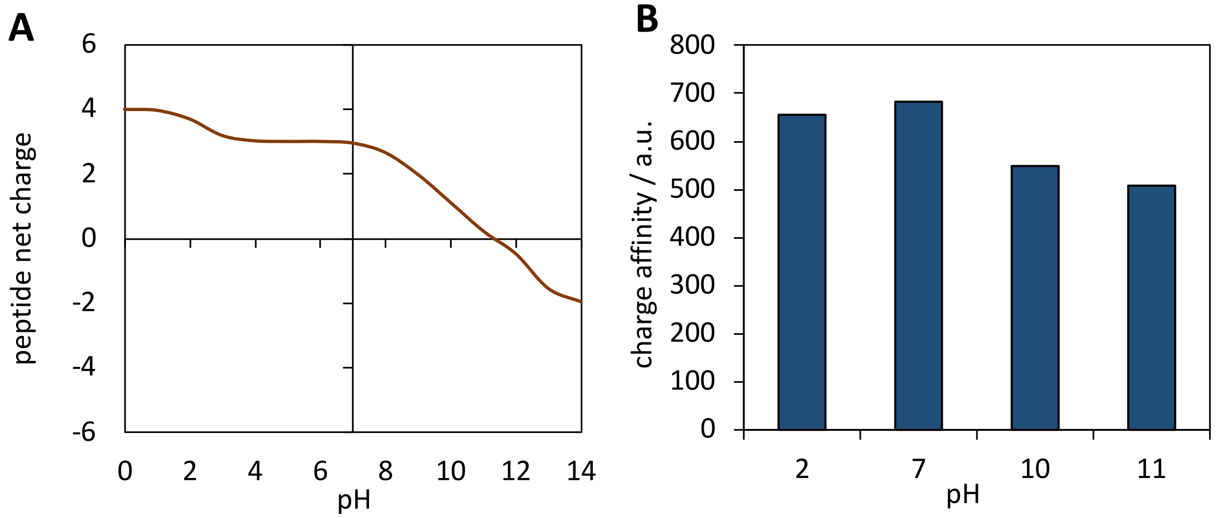

Figure S1. Calculation of peptide properties. A) The net charge of JM#21 is dependent on pH value (data based on peptide calculator tool on www.biosynth.com). B) Charge affinity of the peptide was calculated by scanning with a negatively charged hydroxide probe at varying pH-dependent peptide net charges.

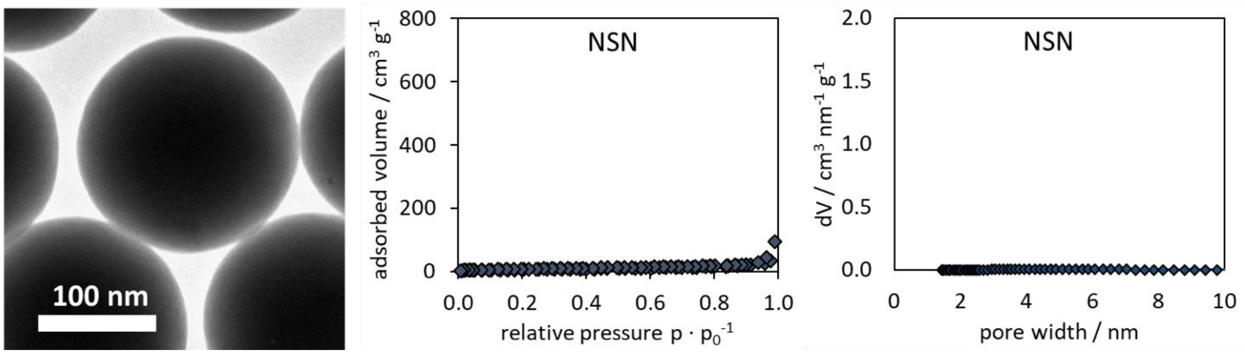

Figure S2. Transmission electron microscopy and nitrogen sorption measurements at 77K of non-porous NSN particle. Pore-size distribution curves were calculated using the equilibrium NLDFT kernel developed for silica in the relative pressure range from 0 to 0.9.

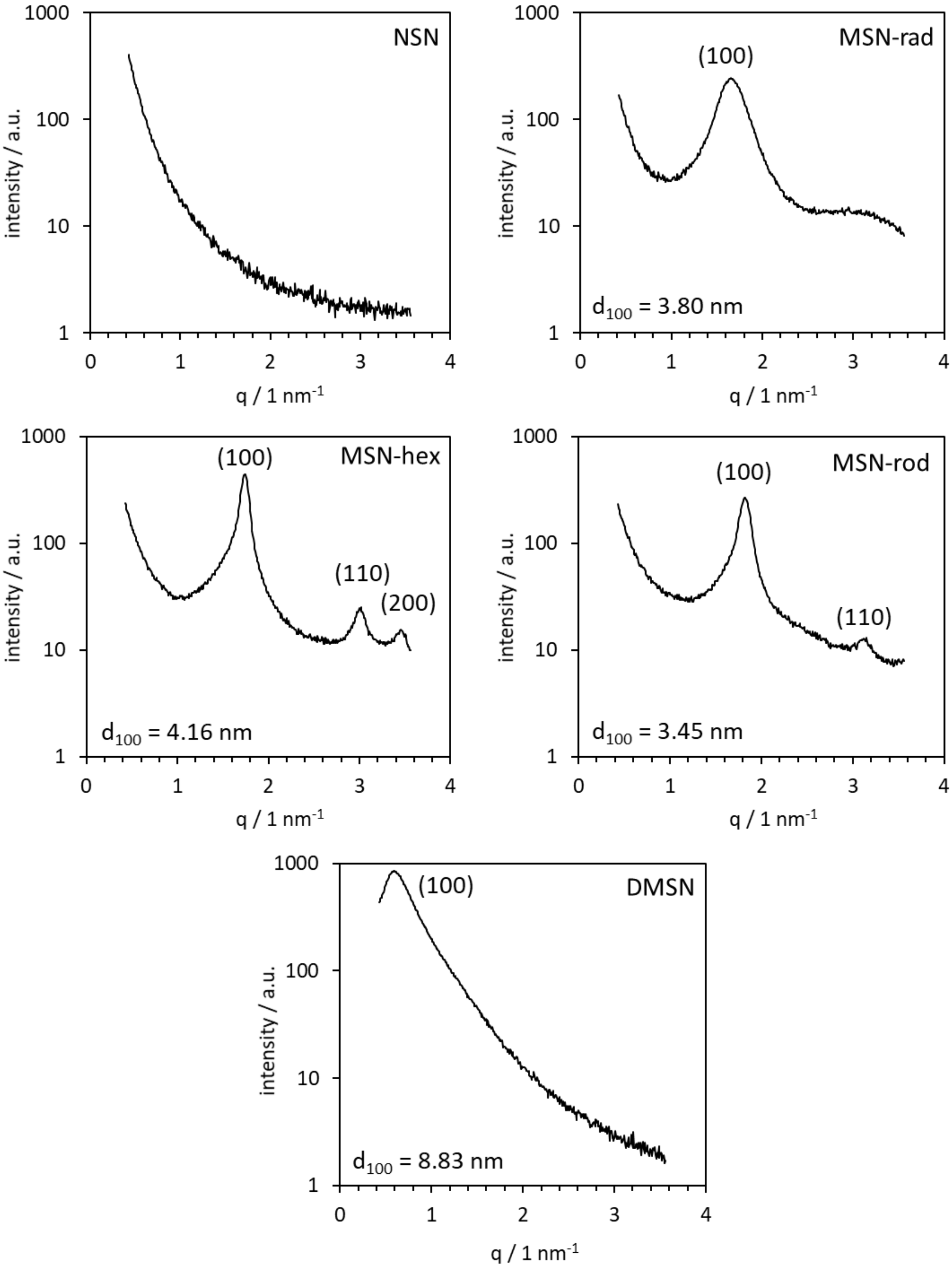

Figure S3. Small-angle x-ray scattering (SAXS) measurement of NSN, MSN-rad, MSN-hex, MSN-rod and DMSN. Calculated d-spacings (d_{100}) at the first order maximum are indicated in the diffractograms.

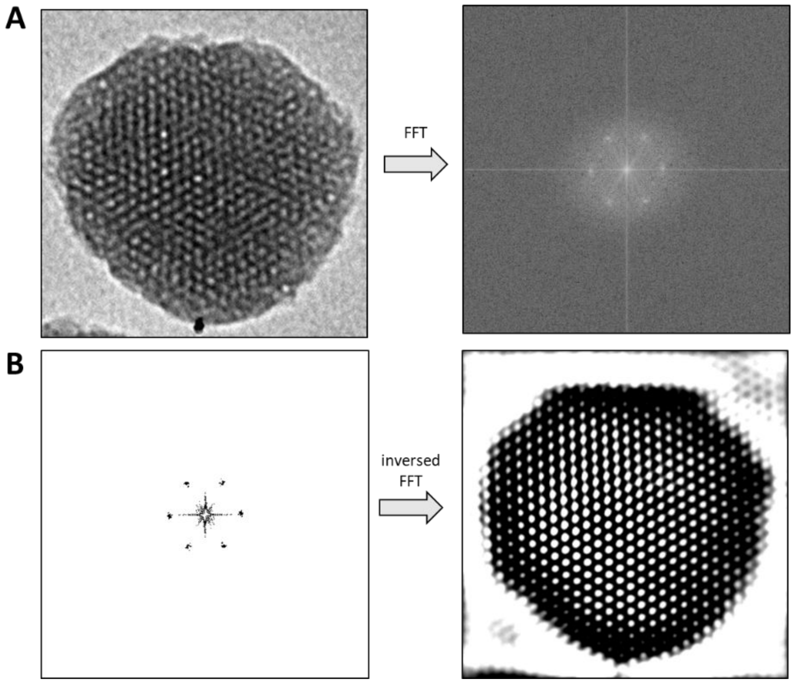

Figure S2. A) fast Fourier transformation (FFT) of a representative TEM image of MSN-hex. B) frequency range filtering of the diffraction pattern and subsequent inverse FFT revealing the electron diffracting pore structures.

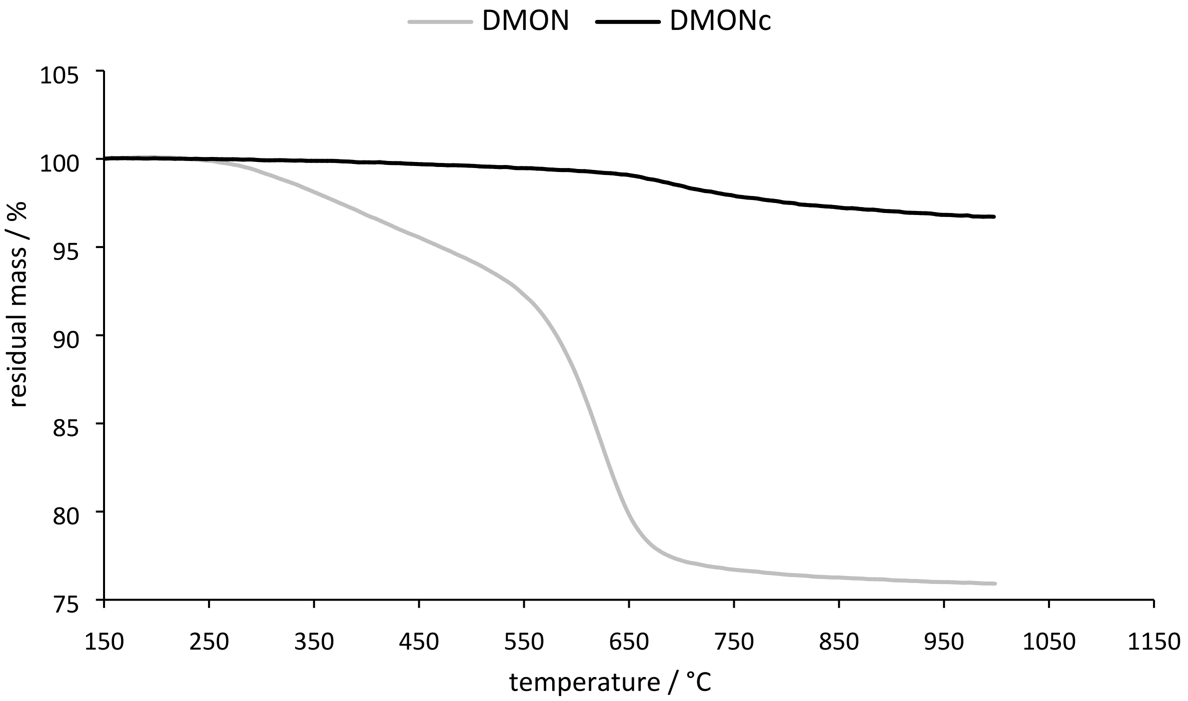

Figure S3. Thermogravimetric analysis (residual mass plots) of samples DMON and DMONc.

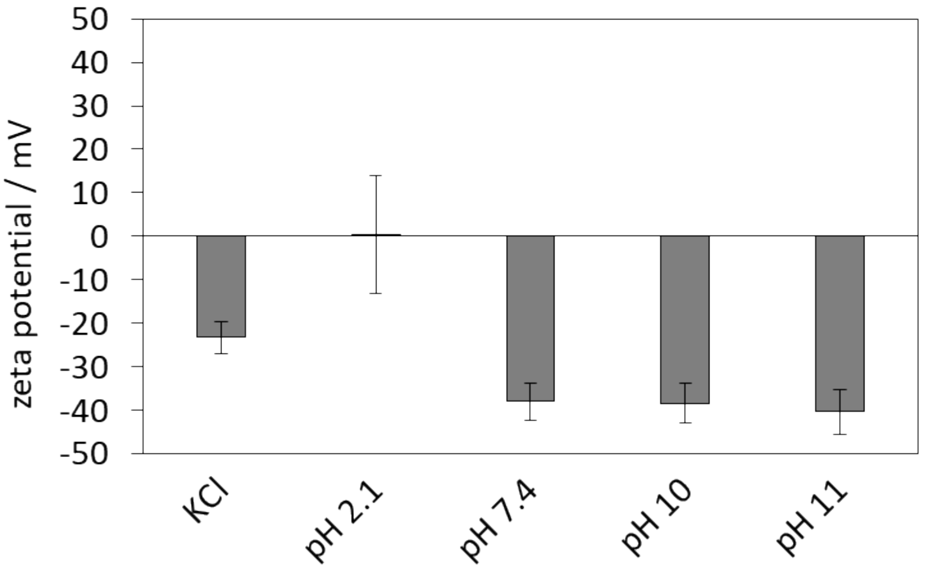

Figure S4. Exemplary zeta potentials of all-silica MSNrad in 1 mM KCl, 12 mM phosphate buffer (pH 2.1 and 7.4) or 12 mM carbonate buffer (pH 10 and 11) (n = 3, mean ± SD).

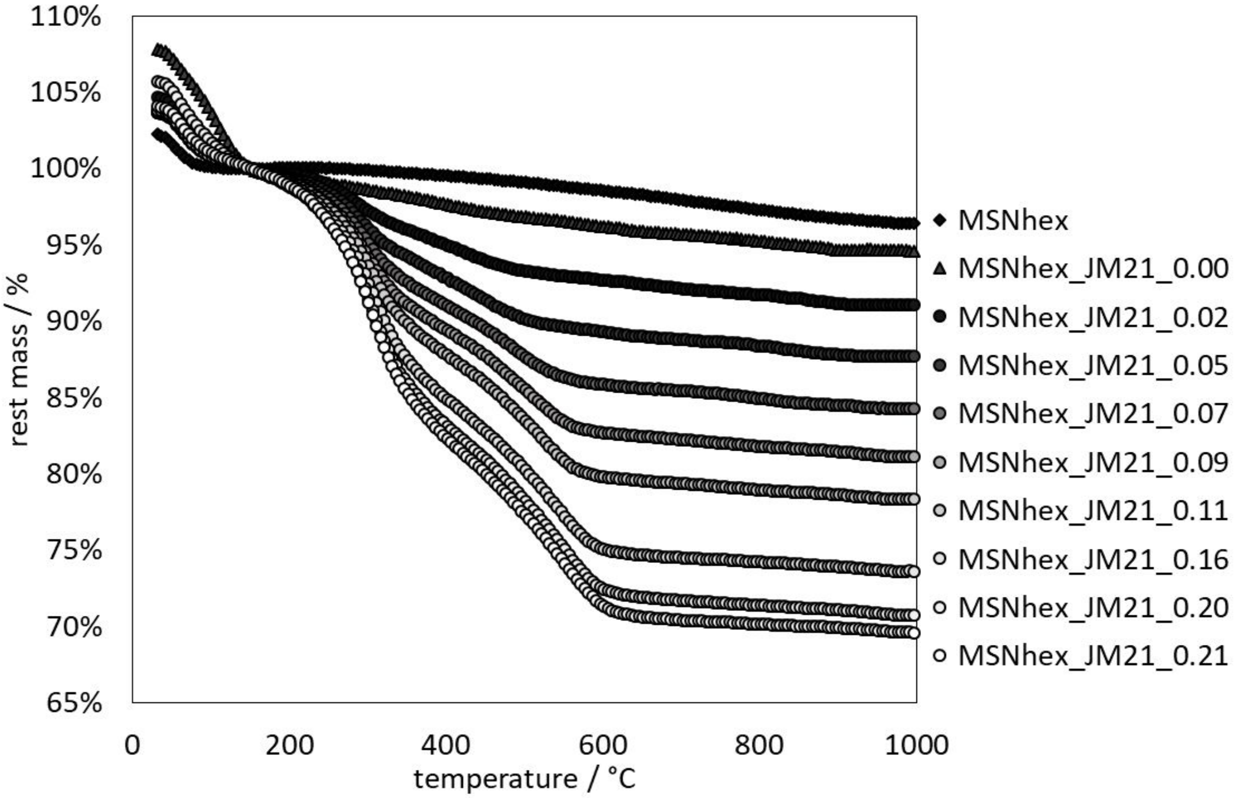

Figure S7. Exemplary thermogravimetric measurements of JM21 loaded MSNhex. The amount of JM21 determined via UV/Vis is indicated in the figure's legend as µmol m^{-2}.

Table S1. Characterization of silica nanoparticles. Specific surface areas were determined by BET analysis of nitrogen sorption measurements at 77 K. The particles' pore diameter and pore volume were calculated by NLDFT analysis for silica (equilibrium model) in the relative pressure range from 0 to 0.9. Zeta potentials were measured in aqueous 25 mM Hepes buffer (pH 7.2). The particle diameter was determined by TEM.

	**MSNrod**	**MSNrad**
diameter / nm	198 ± 26 (length)<br>105 ± 14 (width)	150 ± 13
Surface area / $m^2 \cdot g^{-1}$	1031	986
Pore diameter / nm	3.7	3.2
Pore volume (0.9 $p\ p_0^{-1}$) / $cm^3 \cdot g^{-1}$	0.79	0.65
Zeta-potential / mV	-40 ± 5	-39 ± 4

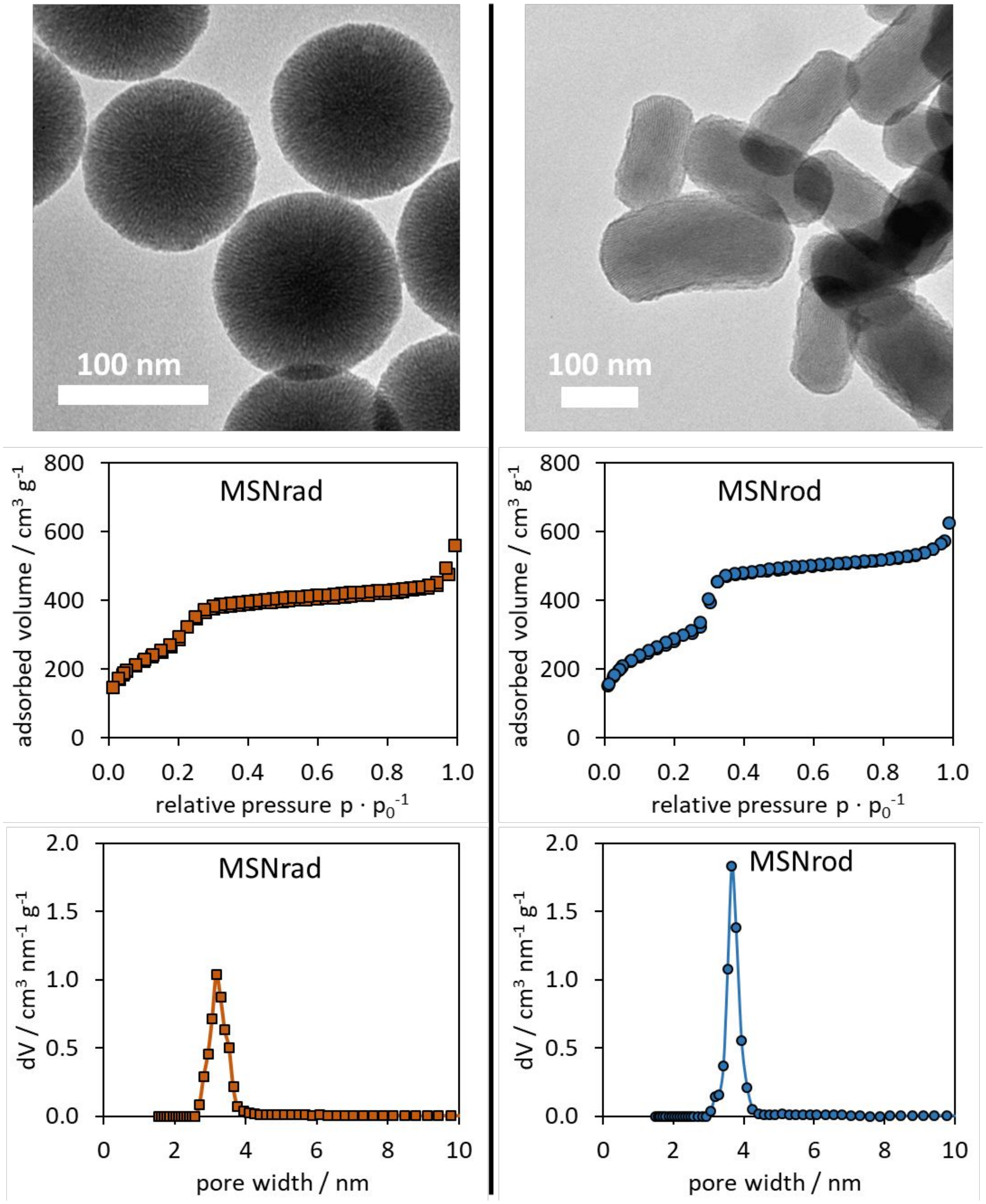

Figure S8. Transmission electron microscopy and nitrogen sorption measurements at 77 K of MSNrad (left) and MSNrod (right). Pore-size distribution curves were calculated using the equilibrium NLDFT kernel developed for silica in the relative pressure range from 0 to 0.9.

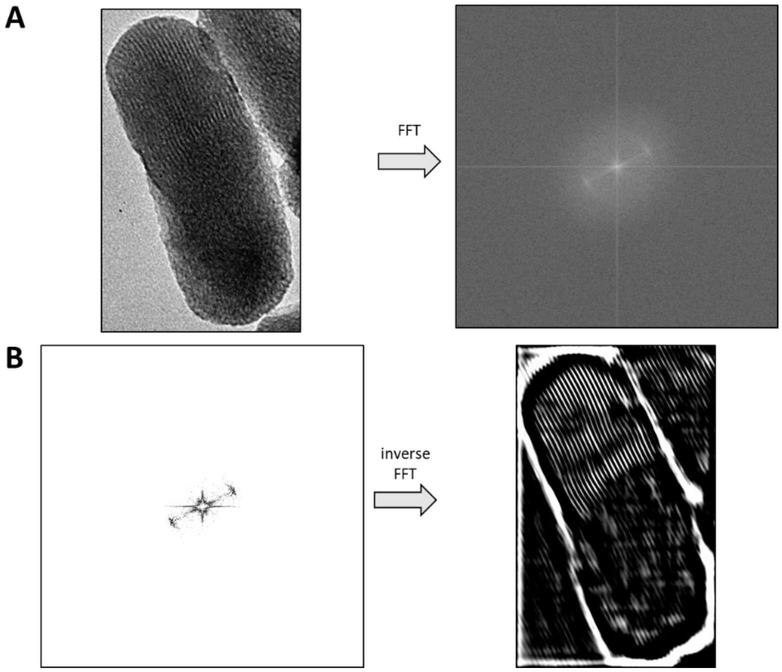

Figure S9. A) fast Fourier transformation (FFT) of a representative TEM image of MSN-rod. B) frequency range filtering of the diffraction pattern and subsequent inverse FFT revealing the electron diffracting pore structures.

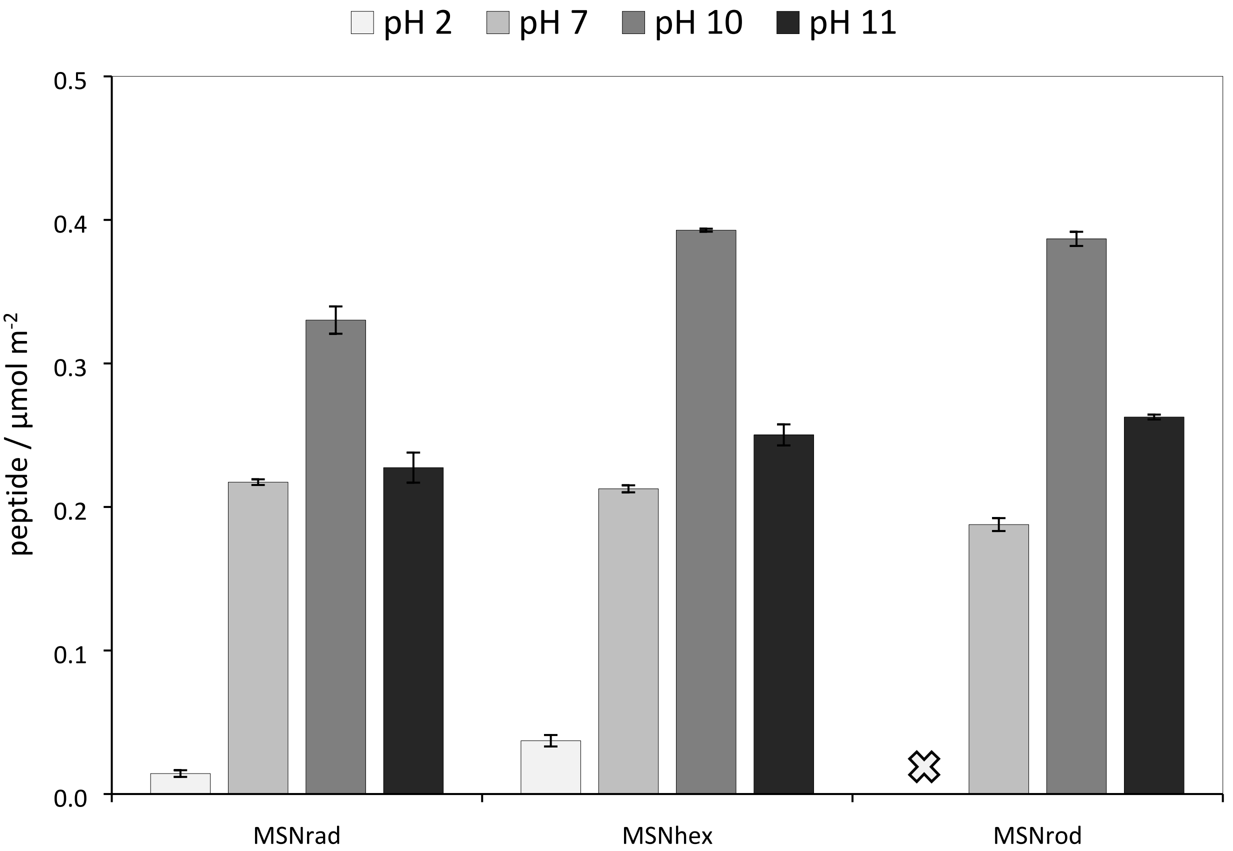

Figure S10. pH-dependent loading degrees for MSNrad, MSNhex and MSNrod. Initial peptide loading concentrations were 2.5 mM at pH 7 (MSNrod: 2 mM), 4 mM at pH 10 and pH 11, respectively (n = 3, mean ± SD).

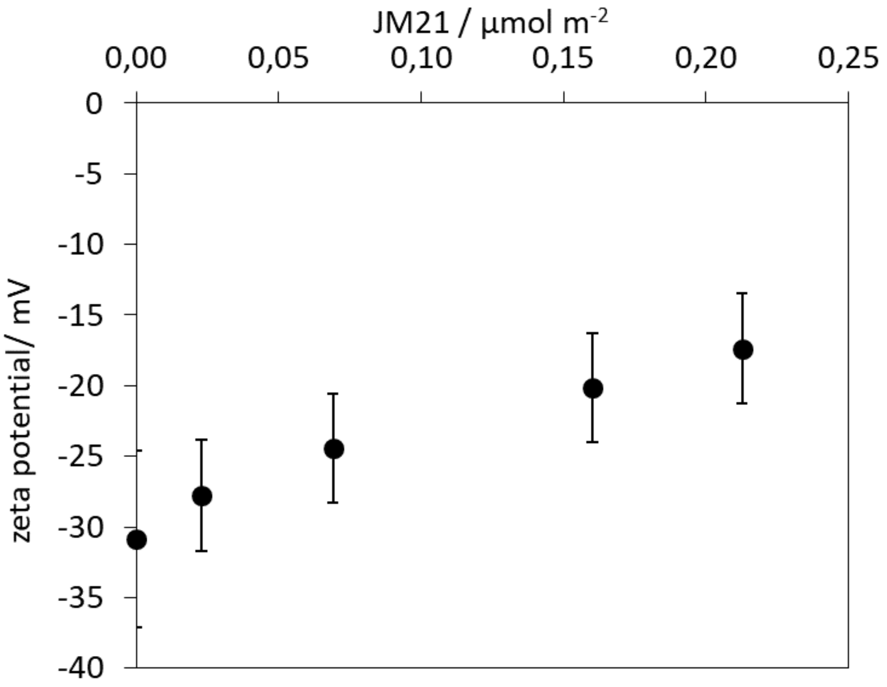

Figure S11. Zeta potential measurements in 25 mM HEPES buffer at pH 7.2 of MSNhex loaded with different amounts of JM#21 (n = 3, mean ± SD).

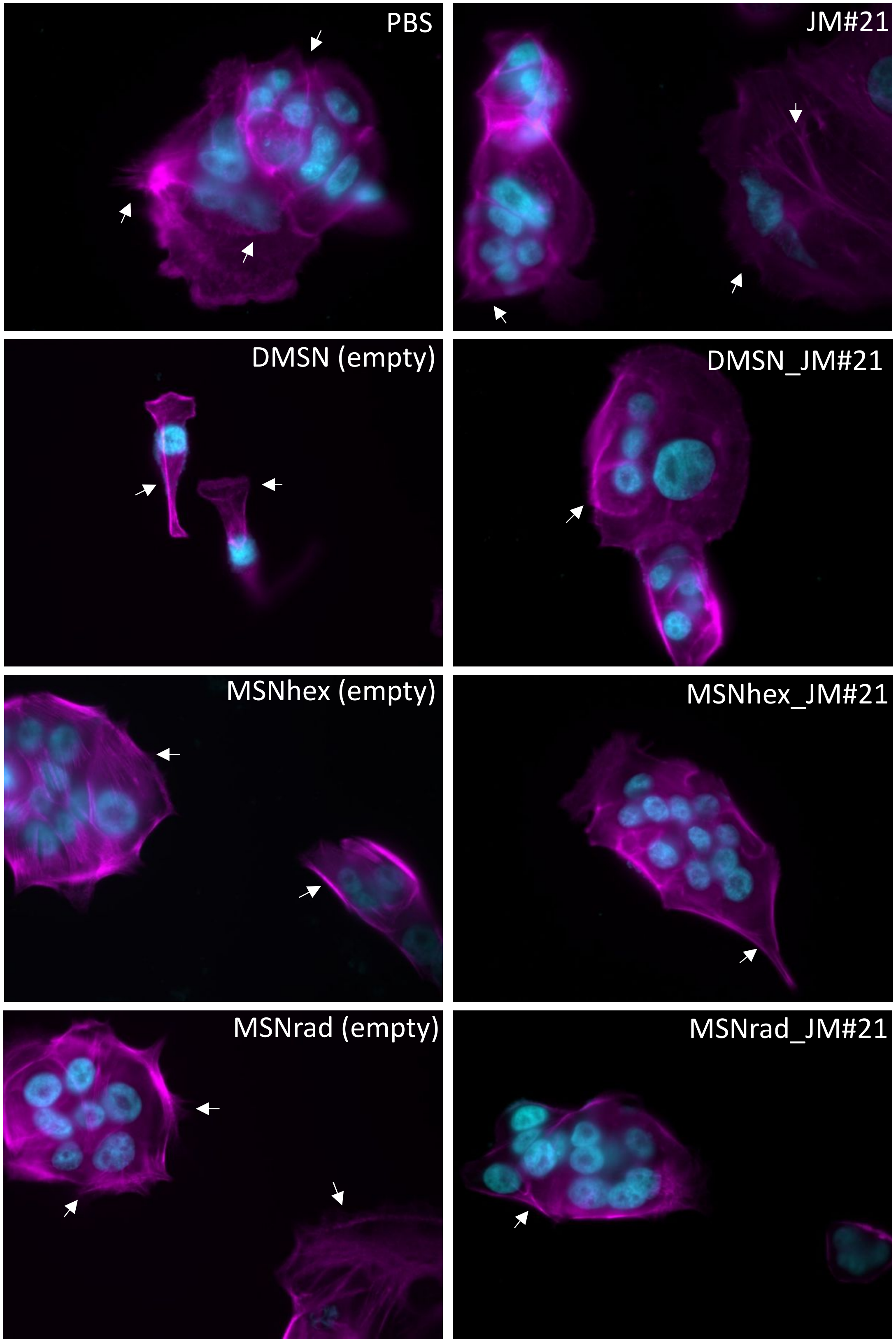

Figure S12. Exemplary fluorescence images of Panc354 treated with CXCL12 (10 µM), together with either PBS, JM#21 (10 µM), non-loaded MSNhex and DMSN (empty) or peptide-loaded MSNhex (30 wt% JM#21)

and DMSN (27 wt% JM#21) in RPMI/10 % FCS for 24 h at 37°C. The particle concentration was normalized to 10 µM of JM#21 at full release. violet: cytoskeleton, blue: cell nuclei, white arrows: indication of mesenchymal-like structural change including stress fibers, spindle like shape of the cell and actin structural changes.

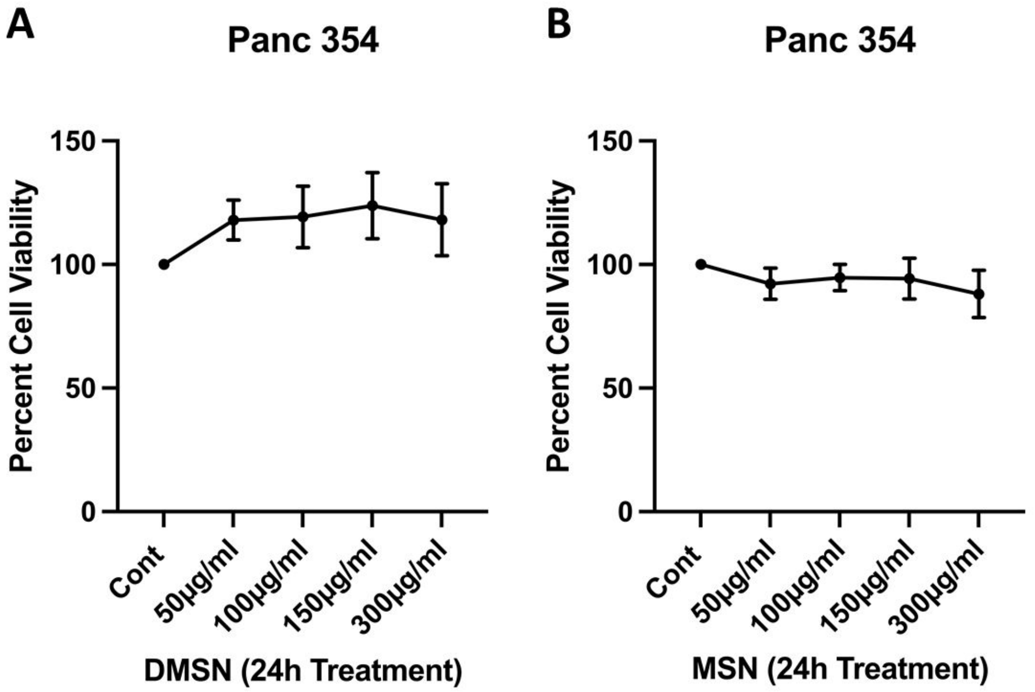

Figure S13. Cell viability measurements of Panc354 cells treated with either A) DMSN or B) MSNhex ("MSN") at different silica concentrations (normalized on residual mass at 600°C) for 24 h at 37°C. Cell culture medium served as control (cont).
